# Identification of EGFR mutation status in male patients with non-small-cell lung cancer: role of ^18^F-FDG PET/CT and serum tumor markers CYFRA21-1 and SCC-Ag

**DOI:** 10.1186/s13550-023-00976-5

**Published:** 2023-04-04

**Authors:** Maoqing Jiang, Ping Chen, Xiuyu Guo, Xiaohui Zhang, Qiaoling Gao, Jingfeng Zhang, Guofang Zhao, Jianjun Zheng

**Affiliations:** 1 Department of Radiology, Ningbo No. 2 Hospital, No. 41 Xibei Street, Haishu District, Ningbo, Zhejiang China; 2 Department of Nuclear Medicine, Ningbo No. 2 Hospital, Ningbo, Zhejiang China; 3 Department of Nephrology, Ningbo No. 2 Hospital, Ningbo, Zhejiang China; 4 Department of Thoracic Surgery, Ningbo No. 2 Hospital, No. 41 Xibei Street, Haishu District, Ningbo, Zhejiang China

**Keywords:** Epidermal growth factor receptor, Non-small-cell lung cancer, Serum tumor markers, ^18^F-2-fluoro-2-deoxyglucose, Positron emission tomography

## Abstract

**Background:**

The high incidence of epidermal growth factor receptor (EGFR) mutations is usually found in female patients with lung adenocarcinoma who have never-smoked. However, reports concerning male patients are scarce. Thus, this study aimed to explore a novel approach based on ^18^F-fluoro-2-deoxy-2-deoxyglucose (^18^F-FDG) PET/CT and serum tumor markers (STMs) to determine EGFR mutation status in male patients with non-small-cell lung cancer (NSCLC).

**Methods:**

A total of 121 male patients with NSCLC were analyzed between October 2019 and March 2022. All patients underwent ^18^F-FDG PET/CT scan before treatment and monitored 8 STMs (cytokeratin 19 fragment [CYFRA21-1], squamous cell carcinoma-related antigen [SCC-Ag], carcinoembryonic antigen [CEA], neuron-specific enolase [NSE], carbohydrate antigen [CA] 50, CA125, CA72-4, and ferritin). A comparison was done between EGFR mutant and wild-type patients in terms of the maximum standardized uptake value of primary tumors (pSUV_max_) and 8 STMs. We performed receiver operating characteristic (ROC) curve and multiple logistic regression analyses to determine predictors for EGFR mutation status.

**Results:**

EGFR mutations were detected in 39 patients (32.2%). Compared with patients with EGFR wild-type, EGFR-mutant patients had lower concentrations of serum CYRFA21-1 (2.65 vs. 4.01, *P* = 0.002) and SCC-Ag (0.67 vs. 1.05, *P* = 0.006). No significant differences of CEA, NSE, CA 50, CA125, CA72-4 and ferritin were found between the two groups. The presence of EGFR mutations was significantly associated with low pSUV_max_ (< 8.75), low serum SCC-Ag (< 0.79 ng/mL) and CYFRA21-1 (< 2.91 ng/mL) concentrations. The area under ROC curve values were 0.679, 0.655, 0.685 and 0.754, respectively, for low CYFRA21-1, SCC-Ag, pSUV_max_ and the combination of these three factors.

**Conclusions:**

We demonstrated that low concentrations of CYFRA21-1 and SCC-Ag, as well as low pSUV_max_, were associated with EGFR mutations, and that the combination of these factors resulted in a higher differentiation of EGFR mutation status in male patients with NSCLC.

**Supplementary Information:**

The online version contains supplementary material available at 10.1186/s13550-023-00976-5.

## Background

The vast majority of cancer-related deaths are caused by lung cancer, which accounts for 21% of all cancer deaths in the USA in 2022 [1]. Non-small-cell lung cancer (NSCLC) is the main type of lung cancer, accounting for about 85% of the total number of patients with lung cancers [2]. Due to the introduction of tyrosine-kinase inhibitors (TKIs), the treatment of NSCLC, especially advanced adenocarcinoma (ADC), has undergone a significant paradigm shift. Epidermal growth factor receptor (EGFR) mutations are the most common druggable targets in patients with NSCLC [3]. The effectiveness of TKIs depends on the presence of EGFR mutations, and patients with NSCLC receiving TKIs therapy have a longer progression-free survival (PFS) than those receiving chemotherapy alone [4]. Based on these discoveries, molecular profiling is recommended for patients with advanced NSCLC [5]. However, in most patients sufficient good-quality tumor tissues are unable to obtain for gene alteration analyses.

Epidemiological studies investigated the difference in clinical characteristics between EGFR mutant and EGFR wild-type NSCLC patients, which showed that EGFR mutations were significantly associated with female, never-smokers and lung ADC [6–9]. However, a relatively high incidence of EGFR mutations was also observed in male patients who smoked with ADC [10]. Thus, the EGFR mutation tests should not be ignored concerning these populations, but the number of reports is very small.

^18^F-2-fluoro-2-deoxyglucose (FDG) positron emission tomography/computed tomography (PET/CT), a molecular imaging device that reflects metabolic features, is widely used in the diagnosis and staging of lung cancer [11, 12]. The relationship between EGFR mutation and metabolic activity of lung cancer has been evaluated. However, contradictory results were observed [11, 13, 14]. In terms of predicting EGFR mutation status in NSCLC, the maximum of standardized uptake value (SUV_max_) of primary lesions from ^18^F-FDG PET/CT showed moderate predictive efficacy [14]. Kim et al. showed that the metabolic activity in localized lung ADC with EGFR mutations was low [13]. These findings confirm the applicability of using metabolic parameters to estimate EGFR mutations in NSCLC patients, although the results remain unsatisfactory.

A range of serum tumor markers (STMs) are used clinically for NSCLC screening and response and recurrence monitoring, e.g., carcinoembryonic antigen (CEA), squamous cell carcinoma antigen (SCC-Ag), cytokeratin 19 fragments (CYFRA 21-1), neuron-specific enolase (NSE) and ferritin [15–17]. The associations between EGFR mutations status and serum concentrations of CEA, SCCA and CYFRA21-1 have been assessed. In general, EGFR mutations were more prevalent in patients with high concentration of CEA and low concentrations of CYFRA21- 1 and SCC-Ag [18–20]. However, conflicting reports were also observed [17, 21].

Although EGFR mutations are more common in women, never-smokers, and patients with lung ADC [21], stratification of male patients who are prone to EGFR mutations is also required. Based on the above results, we hypothesized that the metabolic activity of pulmonary lesions on ^18^F-FDG PET/CT, the concentrations of serum tumor markers may contribute to the identification. Therefore, in the present study, we aimed to investigate factors of ^18^F-FDG PET/CT and STMs that correlated with EGFR mutations in male patients with NSCLC.

## Material and methods

### Study design and patient population

**Fig. 1:**
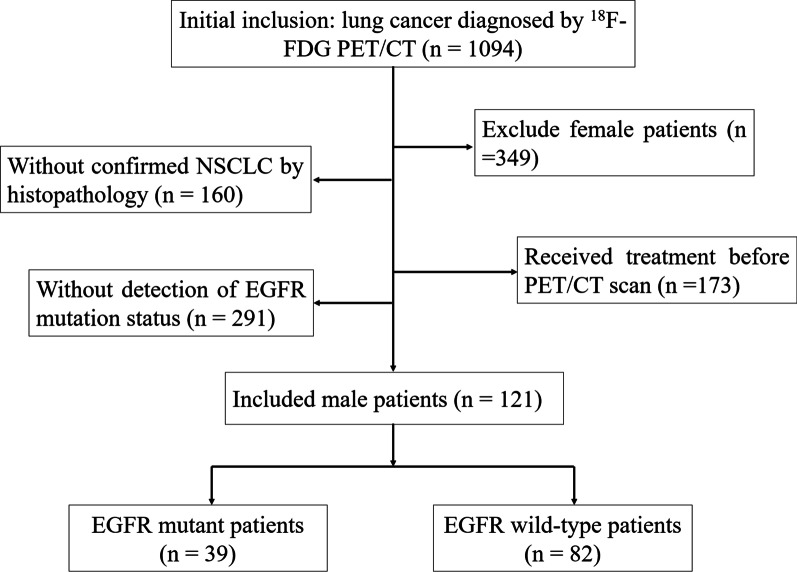
Study design and patient selection algorithm

From October 2019 to March 2022, we studied 1094 consecutive patients who were initially diagnosed with lung cancer using ^18^F-FDG PET/CT at Ningbo No.2 Hospital (Ningbo, China). In order to be eligible for participation in this study, patients had to meet the following four criteria: (1) male patients; (2) there was histopathological confirmation of NSCLC (including ADC, squamous cell carcinoma [SCC], and not otherwise specified [NOS]); (3) no treatment was administered before undergoing ^18^F-FDG PET/CT; (4) EGFR mutation status was detected. Finally, a total of 121 male patients were enrolled in the study after applying the inclusion criteria (Fig. 1). We summarized the clinical characteristics of the study participants, including their age, clinical TNM stages, smoking status, and histopathological subtypes in Table 1. Those known to be never-smokers were defined as having never smoked more than 100 cigarettes during their lifetimes [22].

**Table 1:** Comparison of clinical features and qualitative analysis of serum tumor markers between EGFR wild-type and mutant-type male patients with NSCLC

Characteristics	Total	EGFR		*P* value
		Wild-type	Mutant-type	
Age, years				0.405
Median	67	67	68	
Range	35–85	35–81	44–85	
Smoking history				0.001*
Never-smoker	48 (39.7)	24 (50)	24 (50)	
Ever-smoker	73 (60.3)	58 (79.5)	15 (20.5)	
Clinical TNM stage				0.170
I–II	53 (43.8)	32 (60.4)	21 (39.6)	
III–IV	68 (56.2)	50 (73.5)	18 (26.5)	
Histopathology				0.001*
ADC	80 (66.1)	45 (56.3)	35 (43.7)	
SCC	33 (27.3)	29 (87.9)	4 (12.1)	
NOS	8 (6.6)	8 (100)	0 (0)	
CEA				0.323
Positive	45 (37.2)	28 (62.2)	17 (37.8)	
Negative	76 (62.8)	54 (71.1)	22 (28.9)	
CYFRA21-1				0.006*
Positive	61 (51.3)	49 (80.3)	12 (19.7)	
Negative	58 (48.7)	32 (55.2)	26 (44.8)	
SCC-Ag				0.058
Positive	36 (30.3)	29 (80.6)	7 (19.4)	
Negative	83 (69.7)	52 (62.7)	31 (37.3)	
NSE				0.999
Positive	3 (2.5)	2 (66.7)	1 (33.3)	
Negative	116 (97.5)	79 (68.1)	37 (31.9)	
CA125				0.845
Positive	52 (43.0)	36 (69.2)	16 (30.8)	
Negative	69 (57.0)	46 (66.7)	23 (33.3)	
CA50				0.144
Positive	15 (12.8)	13 (86.7)	2 (13.3)	
Negative	102 (87.2)	68 (66.7)	34 (33.3)	
CA72-4				0.548
Positive	15 (12.8)	12 (80.0)	3 (20.0)	
Negative	102 (87.2)	70 (68.6)	32 (31.4)	
Ferritin				0.103
Positive	44 (36.4)	26 (59.1)	18 (40.9)	
Negative	77 (63.6)	56 (72.7)	21 (27.3)	

*Indicates significant differences between the comparison groups

### PET/CT scan technique

We performed PET/CT scans using a GE Discovery 710 PET scanner (GE Healthcare, Chicago, IL, USA). PET/CT examinations were performed on patients after they had fasted for ≥ 6 h. Glucose levels were measured and confirmed to be < 7.0 mmol/L prior to injection of 5.2–7.4 MBq/kg of ^18^F-FDG, followed by a PET/CT scan performed 45–60 min later. 140 kV, 10 mA, 0.5 s rotation time, and 40 mm collimation were used for the low-dose CT scan. Afterward, a three-dimensional PET scan was performed at 2.5 min per bed position from skull base to upper thigh, and CT data were used to reconstruct the picture from the iterative algorithm. In order to evaluate the PET, CT, and fusion PET/CT images, the Xeleris Workstation (GE Healthcare) was used to obtain images in the transverse, sagittal, and coronal planes.

### Analysis of PET/CT imaging

In all cases, two senior nuclear physicians (MQJ and QLG) with clinical experience of at least 10 years evaluated the PET and CT images consistently. The SUV_max_ was calculated to measure the uptake intensity of ^18^F-FDG in the lesion; abnormal ^18^F-FDG uptake was defined as metabolic activity exceeding the surrounding background [23]. A two-dimensional region of interest (ROI) was manually drawn around the edges of tumor lesions and placed at the region of the tumor with the highest uptake of ^18^F-FDG. According to this definition, SUV_max_ refers to the peak SUV for the ROI pixel with the highest count. In this formula, SUV = (Radioactive concentration in the ROI [MBq/g])/ (Injected dose [MBq]/patient's total body weight [g]) [23]. During visual qualitative analysis, metastatic lymph nodes were identified if their metabolic activity exceeded that of their background mediastinal blood pool [24].

### Analysis of EGFR mutations

The status of EGFR mutations was detected by histological examination of primary tumors, metastatic lymph nodes or organs that were obtained by surgical resection, fiberoptic bronchoscopy biopsy, or fine-needle puncture. In all cases, the samples were fixed in 10% buffered neutral formalin and embedded in paraffin wax. According to instructions provided by the manufacturer, DNA was extracted from the formalin-fixed paraffin-embedded (FFPE) tissue sections using the QIAamp DNA FFPE Tissue Kit (Qiagen NV, Venlo, Netherlands). The polymerase chain reaction was conducted using a Mx3000PTM real-time PCR system (Stratagene, La Jolla, USA). Amplification-refractory mutation system along with an EGFR 29 Mutations Detection Kit (Amoy Diagnostics, Xiamen) was used to detect the status of EGFR mutations. EGFR mutant tumors were identified if exon mutations were detected; otherwise, wild-type tumors were determined.

### Statistical analysis

Descriptive statistics were used to present demographic patient data. The clinical characteristics of patients with and without EGFR mutations were compared, including age, clinical TNM stage, smoking status (never smoker vs. smoker), and histopathological subtypes (ADC, SCC and NOS), using the chi-squared test and Fisher exact test. A median with an interquartile range (IQR) was presented for serum concentrations of tumor markers and metabolic parameters. Mann–Whitney tests were used to compare the differences in continuous variables (SUV_max_ of the primary lesions [pSUV_max_], metastatic lymph nodes [nSUV_max_] and distant metastasis [mSUV_max_]) and serum tumor marker concentrations between patients with and without EGFR mutations. The parameters or factors significantly different between patients with and without EGFR mutations were used to construct receiver operating characteristic (ROC) curves. In order to evaluate the predicted value for a given criterion, the area under the ROC curve (AUC) was calculated. To predict patients' EGFR mutation status, multiple logistic regression analysis was performed. A two-sided *P* value of < 0.05 was considered statistically significant in all analyses. All statistical analyses and graphs were drawn using GraphPad Prism 9.0 (GraphPad Software, San Diego, CA, USA).

## Results

### Patient characteristics

As shown in Table 1, patient characteristics stratified by EGFR mutation status were summarized. The status of EGFR mutations was tested in all participants, and EGFR mutant-type was identified in 39 (32.2%) of the patients, with a median age of 68 years (range 44–85). Of the 121 male patients enrolled, 121 (100%) were tested for serum CEA, CA125 and ferritin, 119 (98.3%) were tested for CYFRA21-1, SCC-Ag and NSE, and 117 (96.7%) were tested for CA50 and CA72-4. As a result of this study, 66 (54.5%) of the 121 patients developed lymph node metastasis, while 41 (33.9%) developed distant metastasis.

### Association between EGFR mutation status and metabolic parameters

**Fig. 2:**
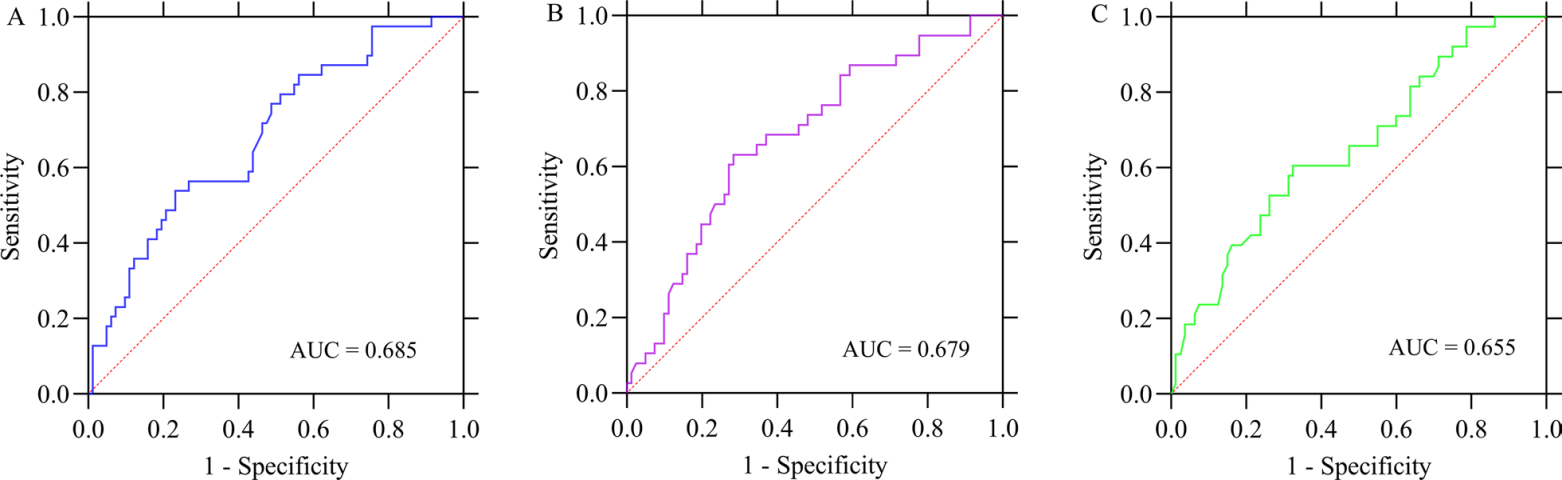
In male patients with NSCLC, the area under the receiver operating characteristic curve (AUC) for individual factors was calculated to predict EGFR mutation status. **A** SUV_max_ of the primary lesions. **B** Serum concentration of CYFRA21-1. **C** Serum concentration of SCC-Ag

**Fig. 3:**
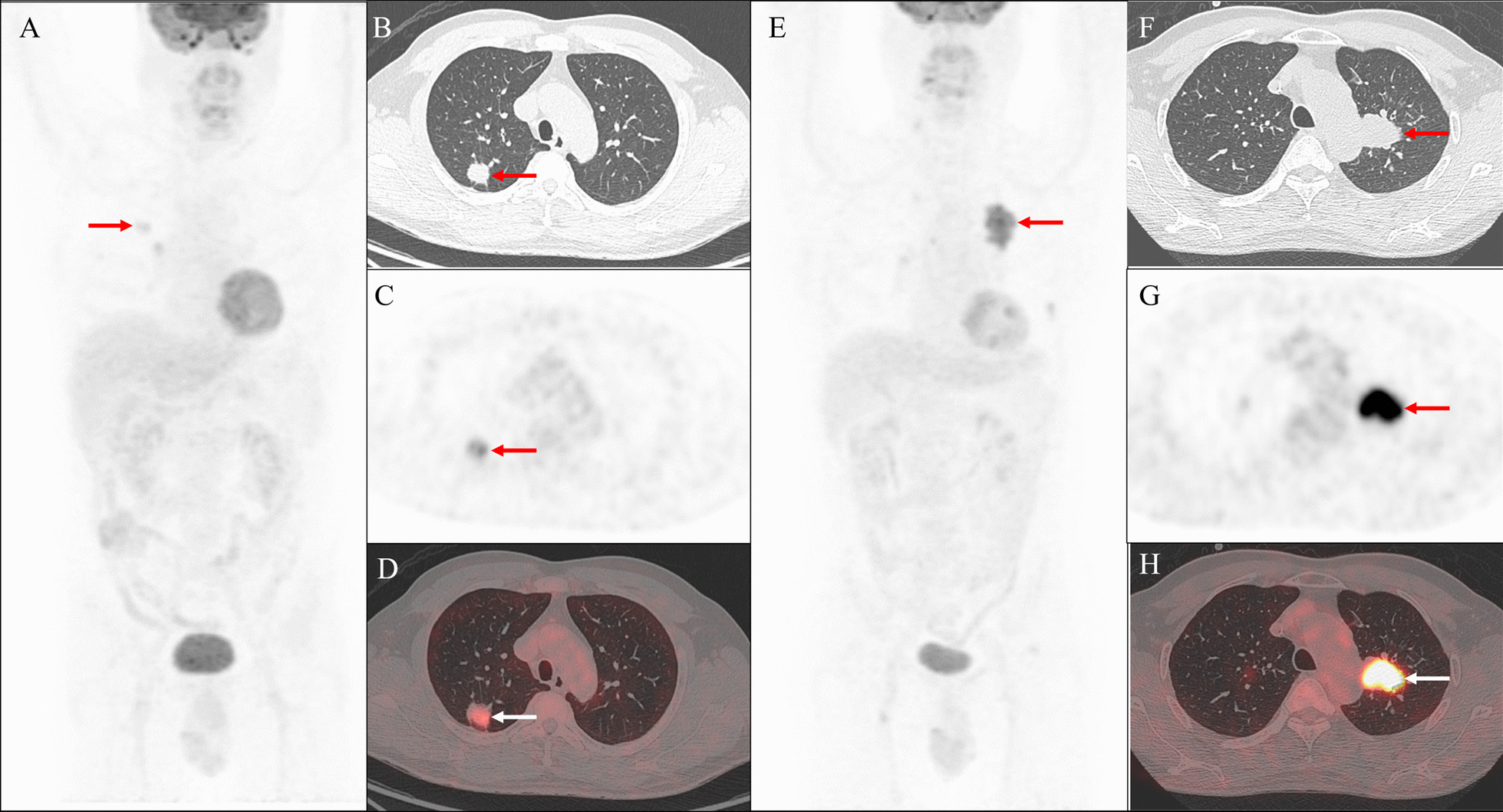
Representative PET/CT images of male patients with EGFR mutation and EGFR wild-type. **A**–**D** A 54-year-old male patients with lung ADC and normal concentrations of serum CYFRA21-1 (0.7 ng/mL) and SCC-Ag (0.58 ng/mL). Three-dimensional maximum intensity projection (3-D MIP) PET images (**A**), axial CT (**B**), PET (**C**), and fusion PET/CT images (**D**) showed a mild increase in ^18^F-FDG uptake of primary lesion (SUV_max_ 6.09) in the right upper lung (arrows). **E**–**H** A 63-year-old male patients with lung ADC and increased concentrations of serum CYFRA21-1 (10.97 ng/mL) and SCC-Ag (4.12 ng/mL). 3-D MIP PET images (**A**), axial CT (**B**), PET (**C**), and fusion PET/CT images (**D**) showed a significantly increase in ^18^F-FDG uptake of primary lesion (SUV_max_ 14.75) in the left upper lung (arrows)

The uptake of ^18^F-FDG could be assessed by SUV_max_. Among 121 male patients, the pSUV_max_ was 11.69 [7.63–15.74], which were subdivided into EGFR-mutant group and EGFR-wild-type group. There was significant difference in pSUV_max_ between the two groups (8.68 [4.98–21.70] vs. 13.01 [8.84–16.44], *P* < 0.001). However, no significant differences of nSUV_max_ (8.41 [5.45–15.77] vs. 8.93 [5.97–12.76], *P* = 0.798) and mSUV_max_ (8.80 [5.96–11.63] vs. 10.78 [5.94–13.93], *P* = 0.365) were observed (Table 2). In addition, ROC curve analysis was performed on pSUV_max_ in order to evaluate its predictive value for the status of EGFR mutations, and when the categorical pSUV_max_ < 8.75, the AUC was 0.685 (Fig. 2A). In Fig. 3, we show representative images of male patients with EGFR mutation and EGFR wild-type, showing their relationship with pSUV_max_ and concentrations of serum tumor markers CYFRA21-1 and SCC-Ag.

**Table 2:** Comparison of metabolic parameters and quantitative serum tumor markers between EGFR wild-type and mutant-type male patients with NSCLC

Factors	Total	EGFR		*P* value
		Wild-type	Mutant-type	
Metabolic parameters (median [IQR])				
pSUV_max_	11.69 [7.63–15.74]	13.01 [8.84–16.44]	8.68 [4.98–21.70]	< 0.001*
nSUV_max_	8.90 [5.93–14.15]	8.93 [5.97–12.76]	8.41 [5.45–15.77]	0.798
mSUV_max_	9.60 [6.05–13.12]	10.78 [5.94–13.93]	8.80 [5.96–11.63]	0.365
Serum tumor markers (median [IQR])				
CEA (ng/mL)	3.06 [1.65–8.77]	2.66 [1.66–8.48]	4.18 [1.73–13.95]	0.286
Ferritin (ng/mL)	205.9 [128.3–387.7]	184.6 [125.1–356.0]	260.6 [135.5–394.9]	0.248
CA50 (U/mL)	9.51 [6.10–14.33]	10.54 [6.49–15.33]	8.73 [4.88–12.13]	0.103
CA125 (U/mL)	12.70 [6.95–32.55]	13.70 [7.25–33.72]	11.40 [5.90–34.20]	0.622
CA72-4 (U/mL)	1.82 [1.00–4.04]	1.94 [1.07–5.98]	1.36 [1.00–2.77]	0.065
NSE (ng/mL)	9.38 [6.72–12.06]	9.38 [6.72–11.51]	9.30 [6.72–12.26]	0.828
SCC-Ag (ng/mL)	0.92 [0.56–1.71]	1.05 [0.63–2.31]	0.67 [0.48–1.44]	0.006*
CYFRA 21-1 (ng/mL)	3.35 [2.39–6.74]	4.01 [2.67–8.16]	2.65 [1.93–4.45]	0.002*

*Indicates significant differences between the comparison groups

### Association between EGFR mutation status and serum tumor markers

We performed qualitative and quantitative analyses of the concentrations of eight STMs according to the status of EGFR mutations. Qualitative analysis showed that the mutation rate of EGFR in negative CYFRA21-1 was higher than that in positive CYFRA21-1 (44.8% vs. 19.7%, *P* = 0.006), while there was no significant difference in EGFR mutations among CEA, CA50, CA125, CA72-4, NSE, SCC-Ag and ferritin (Table 1). Quantitative analysis showed that the concentrations of serum CYFRA21-1 (2.65 [1.93–4.45] vs. 4.01 [2.67–8.16], *P* = 0.002) and SCC-Ag (0.67 [0.48–1.44] vs. 1.05 [0.63–2.31], *P* = 0.006) in patients with EGFR mutant NSCLC were significantly lower than those of the EGFR wild-type, while the remaining serum tumor markers (CEA, CA50, CA125, CA72-4, NSE and ferritin) revealed no significant difference between the two groups (Table 2). In addition, the ROC curves of CYFRA21-1 and SCC Ag were analyzed to evaluate their predictive value for EGFR mutations. When classified as CYFRA21-1 < 2.91 ng/mL and SCC-Ag < 0.79 ng/mL, the AUCs were 0.679 and 0.655, respectively (Fig. 2B, C).

### Metabolic parameters combined with serum tumor markers and clinical features to predict EGFR mutation status

**Fig. 4:**
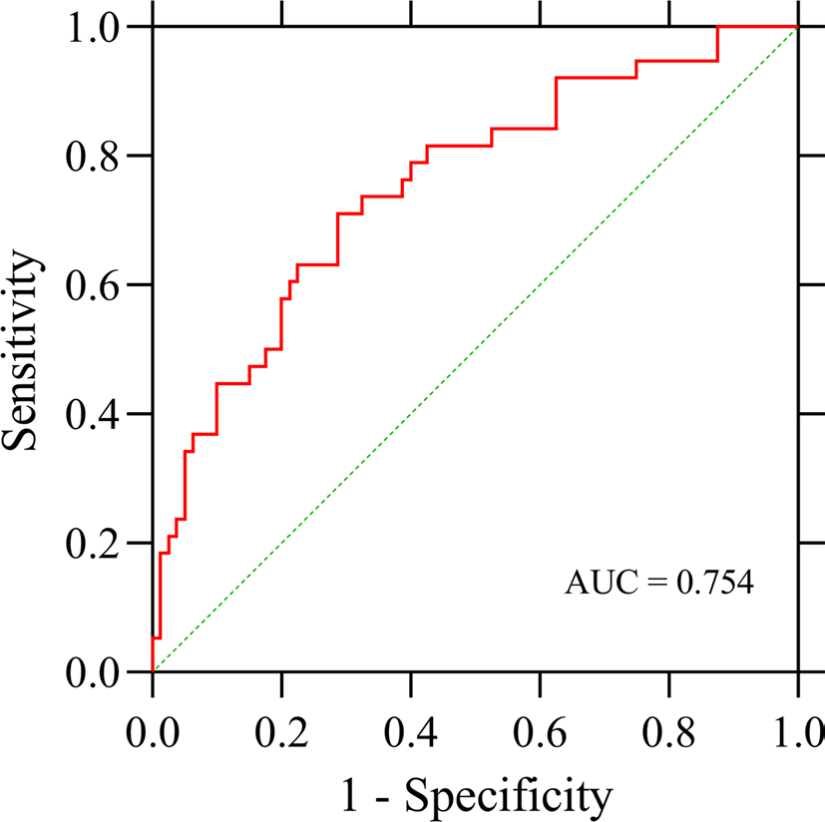
ROC curve for predicting EGFR mutation status in male NSCLC patients using pSUV_max_ and serum tumor markers CYFAR21-1 and SCC-Ag. When pSUV_max_, serum CYFAR21-1 and SCC-Ag concentrations were combined to predict EGFR mutation status, the AUC was 0.754

The univariate logistic regression analysis showed that smoking history, histopathological subtypes, pSUV_max_, CYFAR21-1 and SCC-Ag were significantly associated with the status of EGFR mutations. We, therefore, performed multivariate logistic regression analysis for these factors. Our results showed that when we combined pSUV_max_, concentrations of serum CYFAR21-1 and SCC-Ag to predict EGFR mutation status, the AUC was 0.754 (Fig. 4). When we combined with pSUV_max_, concentrations of serum CYFAR21-1 and SCC-Ag, and smoking history, the AUC was 0.797 (Additional file 1: Fig. 1A). When we combined with pSUV_max_, concentrations of serum CYFAR21-1 and SCC-Ag, and histopathology, the AUC was 0.841 (Additional file 1: Fig. 1B). When we combined these five factors together, the AUC was 0.839 (Additional file 1: Fig. 1C).

## Discussion

In this study, we performed a retrospective analysis using ^18^F-FDG PET/CT and STMs to determine the status of EGFR mutations in male patients with NSCLC. The metabolic parameters, e.g., pSUV_max_, nSUV_max_ and mSUV_max_, and concentrations of eight STMs [CEA, CA50, CA125, CA72-4, NSE, CYFRA21-1, SCC-Ag and ferritin] based on qualitative and quantitative data were analyzed to predict EGFR mutation status. Our findings suggest that the status of EGFR mutations in male NSCLC patients negatively correlated with pSUV_max_, CYFRA21-1 and SCC-Ag, and that a combination of these factors resulted in higher identification of EGFR mutation status in NSCLC patients.

There is evidence that TKIs have a significant therapeutic effect in NSCLC patients with EGFR mutations and prolong their PFS, and overall survival [25–27]. It has been recommended that molecular profiling be the standard of care in patients with advanced NSCLC due to these discoveries [5, 28]. However, in NSCLC patients with advanced disease there is often difficulty in obtaining good-quality tumor tissues for gene alteration analysis. Therefore, a large number of studies with large sample sizes have explored the relationship between EGFR mutation status and clinical characteristics. In a retrospective analysis of 849 Chinese patients, Lv et al. showed that women, non-smokers, adenocarcinoma, and stage I disease were more likely to have EGFR mutations [29]. Wang et al. found similar results in an analysis of 1,089 patients, with EGFR mutations more frequent in females and never-smokers [21]. However, few studies have involved EGFR mutation status in male patients. Sun et al. revealed that gender had no significant effect on the distribution of EGFR mutations in lung adenocarcinoma [30]. Chung et al. showed that the overexpression of P21-activated kinase-1 in lung cancer patients with EGFR mutations may serve as a molecular target, particularly in males [31]. These findings seek to explore new ways to assess EGFR mutation status in male NSCLC patients so that they can benefit from TKIs therapy. However, their further use is limited by the fact that test factors are not routine in clinical applications.

Detection of STMs can assist with the diagnosis of clinically suspected cancer as well as cancer with an unknown primary site [32]. There are currently four best tumor markers available for the management of lung cancer: CEA, SCC-Ag, NSE, and CYFRA 21-1 [21, 33]. Wang et al. showed that negative SCC-Ag and CYFRA 21-1 are associated with EGFR mutation status, and combined gender and histology may enhance the ability of NSCLC patients to distinguish EGFR mutation status [21]. Wu et al. demonstrated that there was a significant correlation between serum ferritin and EGFR mutation status with moderate diagnostic accuracy, and the combination of serum ferritin with CEA improved the diagnostic sensitivity and specificity of EGFR mutation detection in patients with advanced NSCLC [34]. Clinically, rapid, accurate, and low-cost methods are available to detect STMs, and research has previously been conducted about their relationship to EGFR mutation status, although results were inconsistent. In this study, we performed qualitative and quantitative analyses to evaluate the association between STMs and EGFR mutation status. Patients with negative CYFRA21-1 had a higher incidence of EGFR mutations. Moreover, patients with low concentrations of CYFRA21-1 (< 2.91 ng/mL) and SCC-Ag (< 0.79 ng/mL) are associated with EGFR mutation status. Based on quantitative analysis of serum CYFRA21-1 and SC-AG, the AUCs of predicting EGFR mutation status was 0.679 and 0.655, respectively.

^18^F-FDG PET/CT is a widely used molecular metabolic imaging method for diagnosis, staging, and monitoring of treatment response in lung cancer patients [35–37]. The association between metabolic parameters and EGFR mutation status has been investigated in several studies. Low SUV_max_ in the distant metastasis of advanced lung ADC is conducive to the existence of EGFR mutations [24]. There was a considerable variation in the cutoff values of SUV_max_ (7.0–9.91) used to achieve relatively high ROC curve areas (0.557–0.75) [24, 29, 38]. SUV_max_ was not the only parameter used to predict EGFR mutations in NSCLC, but metabolic tumor volume (MTV) was also included. Liu et al. showed that MTV were lower in NSCLC patients harboring EGFR mutations than in patients bearing wild-type EGFR [39]. It was found, however, conversely, that patients with NSCLC harboring EGFR mutations had significantly higher metabolic activity of ^18^F-FDG (e.g., SUV_max_) as compared to patients with wild-type EGFR [40, 41]. Even no significant difference in ^18^F-FDG uptake between EGFR mutant and wild-type NSCLC patients has also been reported [42]. Due to these inconsistent observations, further research is needed to verify these findings. To our knowledge, few reports have focused on male patients to explore differences in metabolic activity between EGFR mutants and EGFR wild-type NSCLC. In this study, we performed a retrospective analysis to identify EGFR mutation status in male patients with NSCLC using metabolic parameters, e.g., pSUV_max_, nSUV_max_ and mSUV_max_. Our results showed that male patients with NSCLC harboring EGFR mutations presented a lower pSUV_max_, but no significant differences were observed in nSUV_max_ and mSUV_max_. Based on pSUV_max_, we obtained an AUC of 0.685 in predicting EGFR mutation status.

Due to the lack of high specificity and sensitivity tumor markers, clinical practice usually employs a combination of detection methods in order to improve diagnostic accuracy. The combination of STMs and ^18^F-FDG metabolic parameters was investigated. Patients with elevated serum CEA levels (≥ 5 ng/mL or ≥ 7 ng/mL) had higher frequency of EGFR mutations [40, 43]. The combination of serum CEA level and pSUV_max_ has a higher predictive value for EGFR mutation than that of single application. However, no significant difference in serum CEA concentration between EGFR mutant and wild-type patients was also observed [21]. Interestingly, we only observed significant differences in serum CYFRA21-1 and SCC-Ag concentrations between EGFR wild-type and mutant patients, but no significant differences were found in serum CEA, CA125, CA50, CA72-4, NSE and ferritin concentrations. Besides, the combination of pSUV_max_ with serum CYFRA21-1 and SCC-Ag had a higher predictive value, with AUC of 0.753, while AUC measured separately was 0.685, 0.679 and 0.655, respectively. Furthermore, when we added smoking history or histopathology to the multivariate logistic analysis, the AUC increased to 0.797–0.841. These findings highlight the significance of ^18^F-FDG uptake, CYFRA21-1, and SCC-Ag serum concentrations in predicting EGFR mutation status in men with NSCLC, with higher predictive values obtained by adding smoking history and histopathology.

Although we found significant value in predicting EGFR mutation status in male NSCLC patients with pSUV_max_, serum CYFRA21-1, and SCC-Ag concentrations, our study had some limitations. Firstly, this was a retrospective analysis and included a small number of patients. Secondly, we aimed to explore the factors related to EGFR mutation status in male patients, where gender bias was innovative but also a limitation of this study. Third, it would be great if we could monitor the therapeutic response of TKIs and jointly assess prognosis based on these factors. Further prospective studies with large sample sizes are needed to validate our findings and explore implications for monitoring treatment response and assessing outcomes.

In summary, we proposed the role of ^18^F-FDG PET/CT and serum tumor markers in recognizing EGFR mutation status in male NSCLC patients. Our results indicate that SUV_max_ of primary lesions and serum CYFRA21-1 and SCC-Ag concentrations are important factors in predicting EGFR mutation status. Compared with EGFR wild-type patients, EGFR mutation patients had lower pSUV_max_ (< 8.75), serum CYFRA21-1 (< 2.91 ng/mL) and SCC Ag (< 0.79 ng/mL) concentrations. Combining these three factors, the AUC for predicting EGFR mutation was 0.753, which was of moderate predictive value. In addition, if we further combine smoking history or histopathological examination, we will obtain a higher predictive value. However, further prospective studies are warranted to validate our findings.

## Supplementary Information

**Additional file 1: Fig. S1**. ROC curves for predicting EGFR mutation status in male NSCLC patients using pSUVmax, serum tumor markers (CYFAR21-1 and SCC-Ag) and clinical features. (A) When combination of pSUVmax, concentrations of serum CYFAR21-1 and SCC-Ag, and smoking history; (B) combination of pSUVmax, concentrations of serum CYFAR21-1 and SCC-Ag, and histopathology, (C) combination of all these five factors together, the AUCs were 0.797, 0.841 and 0.839, respectively.

## Data Availability

The datasets used and analyzed during the current study are available from the corresponding author on reasonable request.

## Abbreviations

PET: Positron emission tomography
CT: Computed tomography
^18^F-FDG: ^18^F-2-fluoro-2-deoxyglucose
EGFR: Epidermal growth factor receptor
TKIs: Tyrosine-kinase inhibitors
NSCLC: Non-small-cell lung cancer
ADC: Adenocarcinoma
SCC: Squamous cell carcinoma
NOS: Not Otherwise Specified
ROC: Receiver operating characteristic
AUC: Area under the ROC curve
SUV_max_: Maximum of standardized uptake value
pSUV_max_: Maximum of standardized uptake value of primary tumor
nSUV_max_: Maximum of standardized uptake value of metastatic lymph nodes
mSUV_max_: Maximum of standardized uptake value of distant metastasis
PFS: Progression-free survival
STMs: Serum tumor markers
CEA: Carcinoembryonic antigen
CYFRA21-1: Cytokeratin 19 fragment
SCC-Ag: Squamous cell carcinoma-related antigen
NSE: Neuron-specific enolase
CA 50: Carbohydrate antigen 50
CA125: Carbohydrate antigen 125
CA72-4: Carbohydrate antigen 72-4
ROI: Region of interest
IQR: Interquartile range
ARMS: Amplification-refractory mutation system
