# Supplementary figures and images for: Identification of EGFR mutation status in male patients with non-small-cell lung cancer: role of 18F-FDG PET/CT and serum tumor markers CYFRA21-1 and SCC-Ag
Source: EJNMMI Res. 2023 Apr 4;13:27. doi: 10.1186/s13550-023-00976-5 (PMC10073355; doi:10.1186/s13550-023-00976-5)

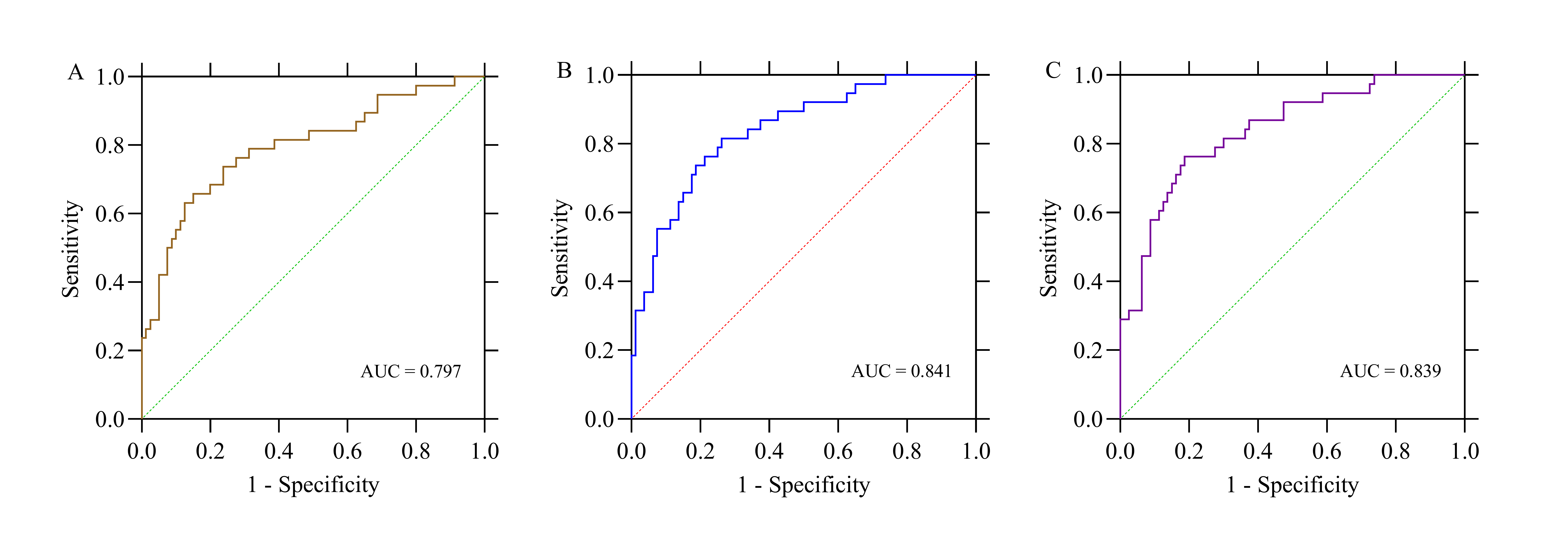

Supplement: Supplementary file 1 — Additional file 1: Fig. S1. ROC curves for predicting EGFR mutation status in male NSCLC patients using pSUVmax, serum tumor markers (CYFAR21-1 and SCC-Ag) and clinical features. (A) When combination of pSUVmax, concentrations of serum CYFAR21-1 and SCC-Ag, and smoking history; (B) combination of pSUVmax, concentrations of serum CYFAR21-1 and SCC-Ag, and histopathology, (C) combination of all these five factors together, the AUCs were 0.797, 0.841 and 0.839, respectively. [file 13550_2023_976_MOESM1_ESM.tif]
